# Supplementary material for: Chromothripsis during telomere crisis is independent of NHEJ, and consistent with a replicative origin
Source: Genome Res. 2019 May;29(5):737–49. doi: 10.1101/gr.240705.118 (PMC6499312; doi:10.1101/gr.240705.118)
Supplement: Supplemental Material [file supp_gr.240705.118_Supplemental_file_1.zip › contigs/annotated_contigs/DB48/contig.2.DB48_length_591_mean_cov_6.26734348562.docx]

**DB48_length_591_mean_cov_6.26734348562**

```
TGTATTTTTACGTTAAGATGAACCAAAATTATTAGACTTATTTAAGATGTACAGGCATCAGAAAAAAGAAGCACATAATGCTTTTGGTG
 >chr7:50471701-50472147 + E=7e-254
CGATGGCACTCACTGTGAACATGTGTAACCACATATTAATATGCAATATTGTTTCCAATACTTTCTAATACAGTTTTTTATAATGTTGT

GTGTGGTGATTGTTCAGGTCGAATCTGTTGTATCCAGTACAGCTTTAGGTCTTCAGCTGCCCTTCTGGCGAGTACATGCACAGGATTGT

AAATGAGAAATGCAGTCATATTTCCAGTCTGCCTCTATGATGATGTTAAATTATTGCTGTTTAGCTGTGAACAAGGGATGTACCACTGG

AGGAATAGAGTATCCTTTTGTACACATTTTGAAATGCTTCTTCTGTAGTGATAGAACAAATAAATGCAACGAATACTCTGTCTGCCC|T

AT|TTTTAGAGGCAGGGCTGCATTGGGAGCATCCAGAACTGCTACCTTCCTAGATGTTTCCCCAGACCGCTGGCTGAGATTCCCTCACC
>chr7:50468513-50468661 + E=2e-77
TGTCGCTTCCTAGAATCCCCTTCTCCAAACGATTAGTCTAAATTTTCAGAGAGAAATAG
```
